# Impact of mevalonate kinase deficiency (MKD) on the quality of life in children and young adults: a national multicentre study

**DOI:** 10.1186/1546-0096-9-S1-P24

**Published:** 2011-09-14

**Authors:** Silvia Federici, Alberto Tomasini, Antonella Meini, Matteo Doglio, G Calcagno, Francesco Zulian, Rita Consolini, Martina Finetti, Luciana Breda, Roberta Caorsi, Laura Obici, Romina Gallizzi, Donato Rigante, Mariolina Alessio, Alberto Martini, Marco Gattorno

**Affiliations:** 1 Gaslini Institute, Genova, Italy; 2 IRCCS Burlo Garofolo, Dipartimento di Pediatria, University of Trieste, Trieste, Italy; 3 Dipartimento di Pediatria, Unità di Immunologia e Reumatologia Pediatrica, Spedali Civili E University Of Brescia, Italy; 4 Sezione di Reumatologia Pediatrica, AOU „G. Martino„, Messina, Italy; 5 Dipartimento A.I. di Pediatria,University of Padua, Padova, Italy; 6 Dipartimento di Medicina della Procreazione e dell'Eta' Evolutiva, Pisa, Italy; 7 Clinica Pediatrica,Divisione reumatologia, Ospedale Policlinico di Chieti, Chieti, Italy; 8 Laboratorio di Biotecnologie, IRCCS Policlinico San Matteo, Pavia, Italy; 9 Divisione di Immunologia e Reumatologia Pediatrica,università di Messina, Messina, Italy; 10 Università Cattolica del Sacro Cuore, Roma, Italy; 11 Dipartimento di Pediatria, Università Federico II, Napoli, Italy

## Background

MKD is an autosomal recessive disease caused by mutations in the mevalonate kinase (MVK) gene.

## Aim

To analyze the long term follow-up and health related quality of life (HRQL) in MKD.

## Methods

MVK gene was analyzed in 950 consecutive patients with periodic fever. 40 MKD patients were identified. Spontaneous disease course was classified as follows: i) resolution (no episodes in the last 6 months), ii) improvement (reduction of more then 30% of fever episodes) iii) stationary iv) worsening (increase frequency of fever episodes or appearance of new major clinical manifestation).The Child Health Questionnaire (CHQ-PF 50) was used to assess the health related quality of life (HRQL). An international sample of 3315 healthy children (52.2% female), with a mean (SD) age of 11.2 (3.8) years constituted the healthy control group.

## Results

Data on follow-up are available for 31 patients. The mean follow-up was 12.9 years (range 2.3-38.2). Steroid on demand was effective in treating fever episodes. 15 patients showed a significant spontaneous reduction of the frequency of fever episodes. Complete resolution was observed in 3 patients. In 9 patients the disease was stable, in 4 worsened. When compared to healthy age-matched individuals, HRQL of MKD patients was generally affected, particularly for global health, general health perception, mental health, parental-impact emotion and self-esteem (p < 0.001).

## Conclusions

Even if a relevant percentage of MKD patient show a spontaneous amelioration of the disease, most of them display a tendency towards a persistence of fever episodes that significantly affect their quality of life.

